# CD44 and CD24 coordinate the reprogramming of nasopharyngeal carcinoma cells towards a cancer stem cell phenotype through STAT3 activation

**DOI:** 10.18632/oncotarget.11113

**Published:** 2016-08-08

**Authors:** Yao-An Shen, Chia-Yu Wang, Hui-Yen Chuang, John Jeng-Jong Hwang, Wei-Hsin Chi, Chih-Hung Shu, Ching-Yin Ho, Wing-Yin Li, Yann-Jang Chen

**Affiliations:** ^1^ Institute of Biochemistry and Molecular Biology, National Yang-Ming University, Taipei 112, Taiwan; ^2^ Department of Life Sciences and Institute of Genome Sciences, National Yang-Ming University, Taipei 112, Taiwan; ^3^ Department of Biomedical Imaging and Radiological Sciences, National Yang-Ming University, Taipei 112, Taiwan; ^4^ Institute of Clinical Medicine, National Yang-Ming University, Taipei 112, Taiwan; ^5^ Department of Otorhinolaryngology, Taipei Veterans General Hospital, Taipei 112, Taiwan; ^6^ Department of Pathology, Taipei Veterans General Hospital, Taipei 112, Taiwan; ^7^ Department of Pediatrics, Taipei City Hospital, Renai Branch, Taipei 106, Taiwan

**Keywords:** nasopharyngeal carcinoma, cancer stem cell, CD44, CD24, STAT3

## Abstract

Cell surface proteins such as CD44 and CD24 are used to distinguish cancer stem cells (CSCs) from the bulk-tumor population. However, the molecular functionalities of CD24 and CD44, and how these two molecules coordinate in CSCs remain poorly understood. We found that nasopharyngeal carcinoma (NPC) cells with high expression of CD44 and CD24 proteins presented with pronounced CSC properties. Accordingly, a subpopulation of NPC cells with co-expression of CD44 and CD24 were specially enriched in high-stage clinical samples. Furthermore, ectopically expressing the epithelial-mesenchymal transition (EMT) regulator Twist was able to upregulate the stemness factors, and vice versa. This indicates a reciprocal regulation of stemness and EMT. Intriguingly, we found that this reciprocal regulation was differentially orchestrated by CD44 and CD24, and only simultaneous silencing the expression of CD44 and CD24 led to a broad-spectrum suppression of CSC properties. Oppositely, overexpression of CD44 and CD24 induced the reprogramming of parental NPC cells into CSCs through STAT3 activation, which could be blunted by STAT3 inhibition, indicating that CD44 and CD24 collaboratively drive the reprogramming of NPC cells through STAT3-mediated stemness and EMT activation. Consequently, targeting of the CD44/CD24/STAT3 axis may provide a potential therapeutic paradigm for the treatment of NPC through repressing CSC activities.

## INTRODUCTION

The main obstacles for cancer treatment are tumor relapse and metastasis. Several studies have suggested these may involve with a rare subset of cancer cells within tumors, the so-called cancer stem cell (CSC) or tumor initiating cell (TIC) [1]. CSCs hold stem cell properties including self-renewal and differentiation [1]. In addition, CSCs are refractory to radiotherapy and chemotherapy and cause relapse even after therapy [2]. Investigating CSCs may provide new treatment avenues and monitoring pathways during cancer progression. Three major approaches, namely side population selection, tumor sphere formation and specific surface marker screening have been used to isolate CSCs in several kinds of tumors [3]. The CSC surface markers also offer a crucial avenue to identify and develop drugs specifically targeting CSCs [3].

Researchers have attempted to isolate nasopharyngeal carcinoma (NPC) CSCs through side population, and specific biomarkers, such as CD44, and CD24 [4–7]. The CD44 is a polymorphic glycoprotein belonging to the family with different glycosylation and alternative splicing forms. It functions as a leukocyte homing receptor and involves in cell adhesion and migration [8]. Standard isoform of CD44 is expressed almost ubiquitously, while splice variants are only expressed in some epithelia and carcinomas. Interaction of CD44 with its ligand, hyaluronan, will promote tumor growth, strengthen the extravasation and lead to metastasis [9]. Besides, hyaluronan-CD44 interaction was found to enhance the translocation and transcription of the epithelial-mesenchymal transition (EMT) regulator *Twist* [10]. Emerging evidences have indicated that CD44 is a poorer prognostic indicator in several carcinomas. Notably, CD44^+^ cells also exhibit CSC features in head and neck cancers, including NPC [5, 11]. Suppression of the CD44 expression reduced the malignant activities of NPC cell lines [12]. Additionally, CD24 is a highly glycosylated mucin-like antigen on the cell surface. It functions as a B cell marker and involves in the development of B cells and neurogenesis [13]. A high expression level of CD24 has been associated with advanced gastric adenocarcinoma, pancreatic adenocarcinoma, and ovarian carcinoma [14–16]. The emerging evidences depicted that CD24 contributes to some stemness features. CD24 is considered as a CSC surface marker of NPC, pancreatic, and liver tumors [6, 17, 18]. It is particularly worth mentioning that CD24 has been found to be a functional marker to regulate tumor initiation and self-renewal by signal transducer and activator of transcription 3 (STAT3)-mediated Nanog regulation in liver cancer [18].

Epstein-Barr virus (EBV) is closely linked to NPC generation and progression. EBV latent membrane proteins LMP1, LMP2, have also been related to NPC cancer progenitor cells (CPCs) or CSCs [19–21]. However, Kondo et al. reported that LMP1 induced CPCs, but not CSCs in NPC [19]. They found LMP1 would generate NPCs with CD44^high^/CD24^low^ pattern and EMT changes. These CD44^high^/CD24^low^ NPCs manifested reduced expression of stemness genes including *Nanog*, *Oct4*, *Sox2*, and *Nestin*. In addition, these CD44^high^/CD24^low^ NPCs induced by LMP1 did not possess higher percentage of side population cells compared with non-LMP1 infected NPCs [19]. The role of EBV and its relationship with NPC CSC biomarkers, such as CD44 and CD24, need further investigation.

Stemness and EMT changes are two major features frequently presented in CSCs. Several stemness factors, including *Oct4*, *Sox2*, *Klf4*, and *c-Myc*, known as Yamanaka factors, are able to reprogram somatic cells into embryonic stem cell-like cells, the so-called induced pluripotent stem cells (iPSCs) [22]. These stemness genes are also found to be expressed in various CSCs [23–25]. Co-expression of *Oct4* and *Nanog* is able to promote EMT in lung adenocarcinoma [25]. The EMT changes are essential for cancer cell metastasis [26]. Overexpression of EMT regulatory genes, such as *Twist* and *Snail*, imparted normal or neoplastic cells with stemness [27]. Accumulating evidences indicate the association between stemness and EMT [28, 29]. Thus, acquiring stemness and undergoing an EMT might be two important events that contribute to CSC properties. As yet, the mechanism of the crosstalk between stemness and EMT remain unclear.

In this study, we found that NPC CSCs display distinctive surface markers whether they were delivered by behavior selection, overexpressing stemness genes or overexpressing EMT genes. Intriguingly, we found that the CSC surface markers served as mediators in the regulation of both stemness and EMT. It is of critical importance to dissect the biological function of CSC surface markers by which CSC properties are regulated and thereby provide effective strategies for the drug design. Hence, blocking their quintessential pathway as demonstrated in this study should pave a novel way towards the future development of targeted therapy in NPC.

## RESULTS

### CD44^high^/CD24^high^ cells with CSC properties

**Figure 1:**
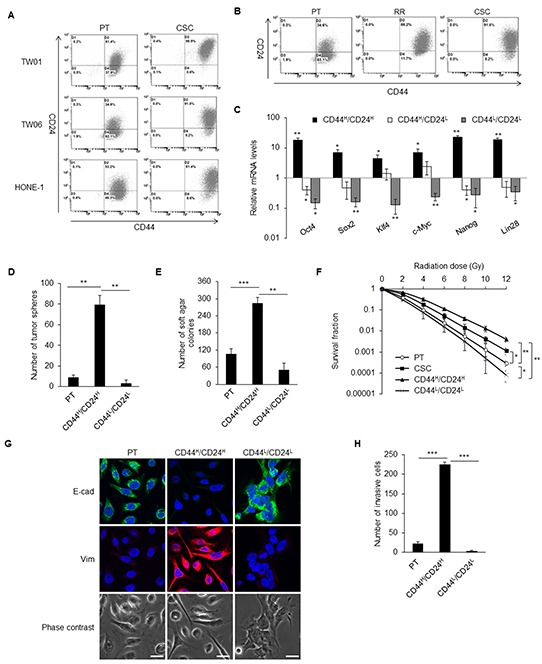
CD44^high^/CD24^high^ cells with CSC properties **A.** The dot plots represent the expression pattern of CD44 and CD24 in three NPC cell lines analyzed by flow cytometry. Left panels are parental cells (PT) and right panels are CSCs. Expression of CD44 and CD24 were higher in the CSCs than in the parental cells in all three NPC lines. **B.** The percentage of CD44^high^/CD24^high^ cells gradually increased during selection process from parental cells (PT), radioresistant clones (RR) to CSCs. The shown data is from TW06 cell line. **C.** The expressions of stemness-associated genes in TW01 CD44^high^/CD24^high^ cells were higher than CD44^high^/CD24^low^ cells, CD44^low^/CD24^low^ cells and the parental cells. Results were got through qPCR analysis, normalized with the mRNA expression level of *18S rRNA* and compared with parental NPC cells. **D.** TW01 CD44^high^/CD24^high^ cells had higher spherogenesis capacity compared with parental cells and CD44^low^/CD24^low^ cells. **E.** TW01 CD44^high^/CD24^high^ cells possessed higher clonogenic formation capacity compared with parental NPC cells and CD44^low^/CD24^low^ cells as revealed by soft agar assay. **F.** Both TW01 CD44^high^/CD24^high^ cells and CSCs exhibited higher radioresistant capacity compared with parental cells and CD44^low^/CD24^low^ cells. **G.** The epithelial type of TW01 parental cells and CD44^low^/CD24^low^ cells and mesenchymal type of TW01 CD44^high^/CD24^high^ cells were differentiated evidently by immunostaining. Scale bars indicate 20 μm. **H.** TW01 CD44^high^/CD24^high^ cells had the greatest invasion ability among parental cells and CD44^low^/CD24^low^ cells. These results are representative of 3 independent experiments. CD44^H^/CD24^H^: CD44^high^/CD24^high^ cells, CD44^H^/CD24^L^: CD44^high^/CD24^low^ cells, CD44^L^/CD24^L^: CD44^low^/CD24^low^ cells. (*: *p*<0.05; **: *p*<0.01; ***: *p*<0.001).

We employed a behavior selection method to isolate NPC CSCs based on the pivotal CSC properties of radioresistance, chemoresistance, and forming spheroid tumor masses [30]. The selected CSCs highly co-expressed CD44 and CD24 in TW01, TW06, and HONE-1 NPC cell lines and showed increment of cells with CD44^+^/CD24^+^ expression from around 30–60% in parental cells to 90% in CSCs (Figure 1A). In these three NPC cell lines, the percentage of CD44^high^/CD24^high^ cells gradually increased as higher ratio of CSC subpopulation from parental NPC cells, radioresistant clones, and CSCs (Figure 1B). This signified that CD44^high^/CD24^high^ could be a potential indicator of the CSC sub-population in NPC cells.

In order to evaluate the characteristics of cancer cells with CD44^high^/CD24^high^ and CD44^high^/CD24^low^, CD44^low^/CD24^low^, these cells were isolated from the parental NPC cell lines. CD44^high^/CD24^high^ cells had elevated expression of stemness genes, including *Oct4, Sox2, Klf4, c-Myc, Nanog,* and *Lin28* compared with CD44^high^/CD24^low^, CD44^low^/CD24^low^, and parental cells (Figure 1C). CD44^high^/CD24^high^ cells formed significantly more tumor spheres than did the parental and CD44^low^/CD24^low^ cells (Figure 1D). CD44^high^/CD24^high^ cells also possessed higher *in vitro* tumorigenicity (Figure 1E) and radioresistant capacity (Figure 1F) compared with the parental and CD44^low^/CD24^low^ cells. The CD44^high^/CD24^high^ cells also showed mesenchymal type morphology with higher expression of vimentin, while CD44^low^/CD24^low^ cells and parental cells presented with an epithelial type and higher E-cadherin expression (Figure 1G). A vigorous invasion capacity of CD44^high^/CD24^high^ cells was also observed (Figure 1H).

To measure the *in vivo* tumorigenicity, various numbers of cells were injected into the sub-renal capsule of NOD/SCID mice. CD44^high^/CD24^high^ cells could form tumor mass with only 100 cells, whereas CD44^low^/CD24^low^ cells could not form any tumor even with 10^4^ cells (Table 1, Supplementary Figure S1A). To demonstrate the *in vivo* self-renewal properties, isolated CD44^high^/CD24^high^ cells and CD44^low^/CD24^low^ cells from previous tumors formed by CD44^high^/CD24^high^ cells were retransplanted to another NOD/SCID mouse. CD44^low^/CD24^low^ cells still could not generate tumors, whereas 100 CD44^high^/CD24^high^ cells regrew secondary tumors (Supplementary Figure S1B).

**Table 1:** Tumor formation ability of parental NPC Cells, CSCs, and sorted cells using CD44 and CD24 surface markers

Cell type	Injected cell number			
	100	500	10^3^	10^4^
PT^a^	0/6^#^	0/6	0/6	1/6
CSC^b^	3/6	5/6	6/6	6/6
CD44^high^/CD24^high^ ^c^	1/6	5/6	5/6	6/6
CD44^low^/CD24^low^ ^c^	0/6	0/6	0/6	0/6

a PT: TW01 parental cells;

b TW01 CSCs isolated from behavior selection;

c Cell sorted from TW01 parental cells;

# Data are presented as number of tumors formed/number of injections. The injection site was sub-renal capsule of NOD/SCID mouse.

**Figure 2:**
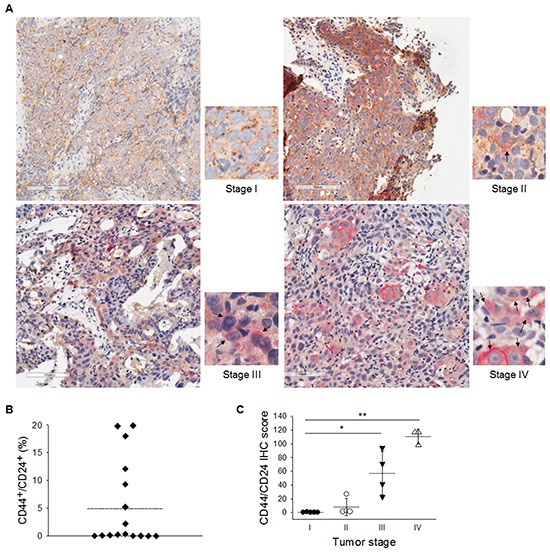
Clinical evidences of CD44^high^/CD24^high^ as a malignancy indicator **A.** Immunohistochemical double-staining of of CD44 and CD24 were performed in primary NPC samples. CD44/CD24 co-expressed cells were detected mainly in stages III and IV NPC sample. CD44 was stained with diaminobenzidene (DAB, brown color) and CD24 with Permanent Red (red color). Hematoxylin was used for nuclear stain. Black arrows indicate cells with co-expression of CD44 and CD24. **B.** The percentage of CD44^+^/CD24^+^ cells in the NPC specimens ranged from 0% to 19.9%. Dotted line indicates the average value. **C.** Increased co-expression of CD44 and CD24 was correlated with the clinical stages of NPC after further scoring. Dotted lines indicate the average values. (*: *p*<0.05; **: *p*<0.01).

To further validate that the CD44^high^/CD24^high^ expression pattern could potentially serve as the CSC biomarker of NPC in clinical samples, tumor cells were sorted from NPC patient's biopsy samples and then xenografted into the NOD/SCID mice. Tumors were found at the injection site of CD44^high^/CD24^high^ cells, whereas CD44^low^/CD24^low^ did not form any tumor (Supplementary Figure S1C). Immunohistochemistry double-staining of CD44 and CD24 was also performed on paraffin-embedded primary NPC samples from patients at different stages of the cancer (Figure 2A). The cells co-expressing CD44/CD24 were scarce at stages I and II (less than 0.34%) but were enriched in the NPC samples at advanced stages, especially at stages III and IV (2.2–19.9%) (Figure 2B). The expression of CD44 and CD24 are highly positive correlation with tumor stage (Figure 2C). These results pinpoint the clinical significance of the surface markers CD44 and CD24 to NPC.

### CD44^high^/CD24^high^ CSCs possessed differentiation potential

The heterogeneity of tumor cells was retained even after metastasis, which might be due to the differentiation potential of CSCs [3]. CD44^high^/CD24^high^ cells asymmetrically gave rise to two distinct daughter groups of cells, the identical CSC like-CD44^high^/CD24^high^ cells as well as the major population of cells, including differentiated cell-like CD44^low^/CD24^low^ cells. This asymmetric division resulted in a decreased in the percentage of CD44^high^/CD24^high^ cells from ~85% to 35% after one week of culture (Supplementary Figure S2A). This indicates that CD44^high^/CD24^high^ cells were able to establish phenotypic diversity and regrow to form a bulk tumor. CD44^low^/CD24^low^ cells, as differentiated cells, merely divided into the same population of differentiated cells that remained to exhibit the CD44^low^/CD24^low^ phenotype after a week (Supplementary Figure S2A). In addition, the long-term self-renewed tumor spheres were enriched in CD44^high^/CD24^high^ cells (Supplementary Figure S2B). After transferring the tumor spheres into a coated-dish, the cells within the spheres started migrating out and underwent differentiation. Cells that migrated out of the tumor spheres gradually lost the expression of CD44 and CD24 (Supplementary Figure S2B).

### EMT and stemness factors generated iCSCs with CD44^high^/CD24^high^ phenotype

**Figure 3:**
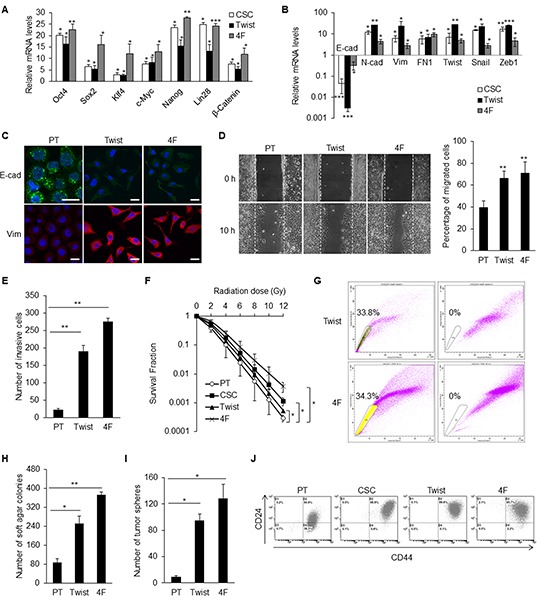
EMT and stemness factors could generate CD44^high^/CD24^high^ iCSCs in NPC **A.** qPCR confirmed the comparable upregulation of stemness genes in TW01 CSCs, Twist-iCSCs, and 4F-iCSCs. Results were normalized with the mRNA expression level of *18S rRNA* and compared with those of the parental NPC cells. Twist: Twist-iCSCs; 4F: 4F-iCSCs. **B.** Comparable upregulation of EMT genes in TW01 CSCs, Twist-iCSCs, and 4F-iCSCs shown by qPCR. Results were normalized with the mRNA expression level of *18S rRNA* and compared with those of the parental NPC cells. **C.** Immunofluorescence staining indicated the repression of epithelial (E-cad) and upregulation of mesenchymal (Vim) markers in TW01 Twist-iCSCs and 4F-iCSCs. **D.** Wound healing assay depicted higher migratory ability of TW01 Twist-iCSCs and 4F-iCSCs compared with the parental cells. Right panel showed the quantified percentage of migrated cells at 10 hours post-wounding. **E.** Transwell cell invasion assay showed higher invasion capacity of TW01 Twist-iCSCs and 4F-iCSCs compared with the parental cells. **F.** TW01 CSCs, Twist-iCSCs, and 4F-iCSCs had higher radioresistance capacity than parental cells. **G.** TW01 Twist-iCSCs had 33.8% and TW01 4F-iCSCs had 34.3% of side population cells. Left dot plot, iCSCs were incubated with Hoechst33342; right plot, iCSCs were pre-incubated with Fumitremorgin C to block the efflux of Hoechst33342. **H.** Soft agar assay indicated that TW01 Twist-iCSCs and 4F-iCSCs had higher clonogenic formation capacity than did the parental cells. **I.** TW01 Twist-iCSCs and 4F-iCSCs exhibited higher tumor sphere formation capacity than did the parental cells. **J.** TW01 CSCs, Twist-iCSCs, and 4F-iCSCs possessed more CD44^high^/CD24^high^ surface antigen phenotype than did the parental cells as measured by flow cytometry. These results are representative of 3 independent experiments. (*, *p*<0.05; **, *p*<0.01; ***, *p*<0.001).

Stemness characteristics can be regenerated by reprogramming of differentiated cells through ectopically expressing some stemness genes or EMT factors [22, 27]. Two kinds of induced caner stem cells (iCSCs) from TW01 NPC cell line were generated. One had an overexpression of *Twist* (hereafter named Twist-iCSCs) and the other overexpressed four stemness cocktail genes, including *Oct4, Sox2, Klf4,* and *c-Myc* (hereafter named 4F-iCSCs). The upregulation of the expression of the stemness genes in Twist-iCSCs and 4F-iCSCs were confirmed by qPCR (Figure 3A). Both iCSCs expressed high levels of EMT regulators like *Twist*, *Snail*, and *Zeb1*, as well as exhibiting an EMT change (Figure 3B and 3C).

Twist-iCSCs and 4F-iCSCs had potent *in vitro* migratory and invasion capacities (Figures 3D and 3E). These iCSCs also exhibited high radioresistant capacity. 4F-iCSCs had the most potent radioresistant capability compared with CSCs and Twist-iCSCs (Figure 3F). Both types of iCSCs had high percentages of side population cells (24.5–34.3%) (Figure 3G). They had high capacities for tumorigenesis and tumor sphere formation *in vitro* (Figure 3H and 3I). The augmented expressions of CD44 and CD24 in both iCSCs were consistent with the prediction of CSC surface markers in NPC (Figure 3J).

### Knockdown of CD44 and CD24 suppressed CSC properties in NPC

**Figure 4:**
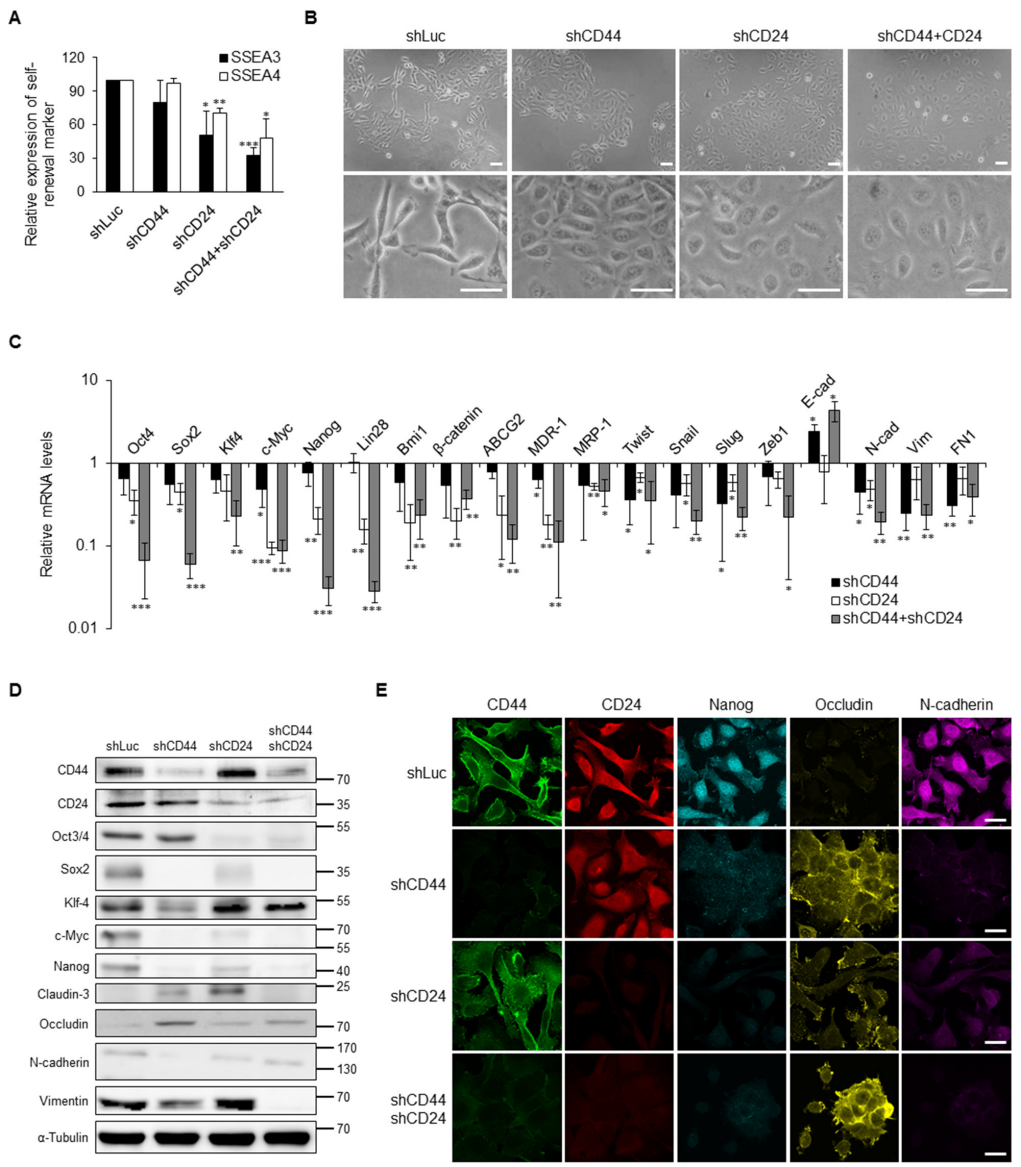
Suppression of stemness and EMT expression via knockdown of CD44 and CD24 in NPC CSCs **A.** CD44 and CD24 knockdown resulted in the decrease of SSEA3 and SSEA4 expression in TW01 CSCs as measured by flow cytometry. **B.** TW01 CSCs underwent mesenchymal-to-epithelial transition after CD44 and CD24 knockdown. Lower panals are 3.5-fold magnified images for a closer look of indicated cells. Scale bars indicate 50 μm. **C.** Knockdown of CD44 and CD24 altered the expression levels of indicated genes measured by qPCR in TW01 CSCs. Results were normalized with the mRNA expression level of *18S rRNA* and normalized to TW01 CSCs transfected with shLuc. **D.** Depletion of CD44 and CD24 suppressed the stemness and EMT markers in TW01 CSCs as measured by western blot. α-tubulin served as internal control. **E.** Knockdown of CD44 and CD24 hindered the stemness and EMT markers in TW01 CSCs as measured by immunofluorescence staining. Scale bars indicate 20 μm. These results are representative of 3 independent experiments. shLuc: cells infected with shRNA-luciferase (shLuc) construct as the negative control. (*: *p*<0.05; **: *p*<0.01; ***: *p*<0.001).

The expression levels of CD44 and CD24 were significantly increased in NPC CSCs and advanced NPC clinical samples. To dissect the biological functions of CD44 and CD24, we used lentivirus-based shRNA to silence the expression of CD44 and CD24 of TW01 CSCs or iCSCs. The self-renewal markers SSEA3 and SSEA4 were significantly down-regulated under CD44 and/or CD24 knockdown condition (Figure 4A, Supplementary Figure S3A). We also noted that cell morphology of CSCs was changed from a spindle-like mesenchymal type to a cobblestone-like epithelial type after knockdown of CD44 and CD24 (Figure 4B). The expression of several stemness genes and EMT markers were also suppressed after blocking the expression of CD44 and CD24 in CSCs (Figures 4C–4E, Supplementary Figure S3B).

**Figure 5:**
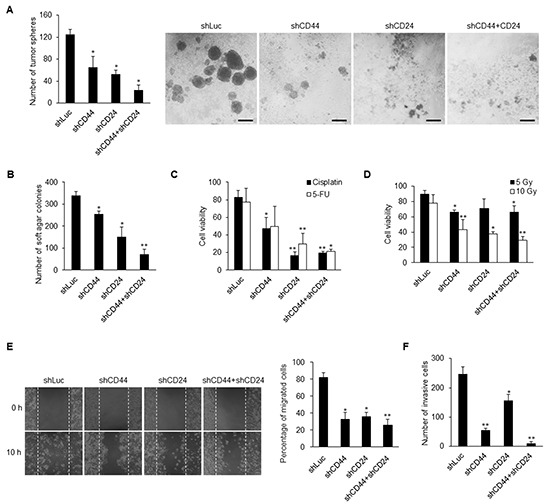
Knockdown of CD44 and CD24 suppresses CSC behavors **A–B.** Knockdown of CD44 and CD24 resulted in decrease of (A) tumor sphere formation and (B) colony formation in TW01 CSCs. Scale bars indicate 100 μm. **C–D.** The sensitivities of cancer cells to (C) chemicals (8 μM cisplatin; 10 μM 5-fluorouracil, 5-FU) and to (D) radiation (5 Gy and 10 Gy) were increased in TW01 CSCs after knockdown of CD44 and CD24 as measured by cell viability assay. **E.** Wound healing assay showed that CD44 and CD24 depletion suppressed the migration capacity of TW01 CSCs. Right panel depicted the quantified percentage of migrated cells at 10 hours post-wounding. **F.** Transwell invasion assay revealed the reduction of invasion capacity of TW01 CSCs with CD44 and CD24 knockdown. These results are representative of 3 independent experiments. (*: *p*<0.05; **: *p*<0.01).

We then examined the significance of CD44 and CD24 in CSC behaviors. The results showed that knockdown of CD44 and CD24 significantly reduced the spheroid formation in the suspension environment and colony formation in soft agar (Figures 5A and 5B, Supplementary Figures S4A and S4B). In addition, we used cisplatin and 5-fluorouracil (5-FU) to verify the drug resistance of NPC cells. We found that CD44 and CD24 co-knockdown CSCs were most sensitive to cisplatin and 5-FU than control CSCs and CD44 or CD24 single-knockdown CSCs (Figure 5C and Supplementary Figure S4C). Furthermore, CD44 and CD24 co-knockdown CSCs exhibited significant sensitivity to irradiation (Figure 5D and Supplementary Figure S4D). Loss of the expression of CD44 and CD24 also repressed the migration and invasion capacity of NPC cells (Figures 5E and 5F, Supplementary Figure S4E). Consequently, CD44 and CD24 are essential in maintaining CSC properties.

### CD44 and CD24 overexpression reprogrammed NPC cells

**Figure 6:**
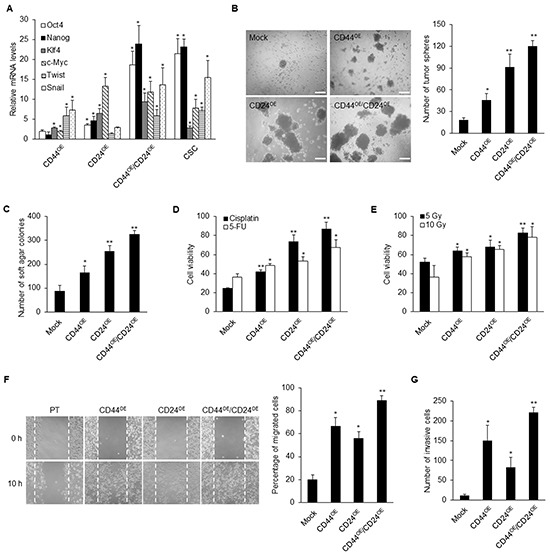
Ectopic expressions of CD44 and CD24 reprogram NPC parental cells **A.** Stemness genes including *Oct4, Nanog, Klf4* and *c-Myc*, as well as EMT-related genes such as *Twist* and *Snail*, were up-regulated in CD44 or CD24 or co-overexpressed TW01 cells when compared with parental cells as determined by real-time RT-PCR. Results were normalized with the mRNA expression level of *18S rRNA* and normalized to mock-transfected TW01 parental cells. **B.** The number of tumor spheres in CD44/CD24 co-overexpressed TW01 cells was higher than in CD44 or CD24 single overexpressed cells and parental cells. Left images depict the sphere morphology of indicated cells. Scale bars indicate 200 μm. **C.** The number of soft agar colonies in CD44/CD24 co-overexpressed TW01 cells was higher than in CD44 or CD24 single overexpressed cells and parental cells. **D–E.** The numbers of viable cells in CD44/CD24 TW01 co-overexpressed cells were significantly increased under (D) cisplatin or 5-FU treatment or (E) irradiation treatment. **F–G.** CD44/CD24 co-overexpressed TW01 cells possessed the highest (F) migration capacity and (G) invasive capacity compared with other groups. Right panel shown in (F) indicated the quantified percentage of migrated cells at 10 hours post-wounding. These results are representative of 3 independent experiments. Mock: TW01 parental cells with mock-infected; CD44^OE^: TW01 parental cells with CD44 overexpression; CD24^OE^: TW01 parental cells with CD24 overexpression; CD44^OE^/CD24^OE^: TW01 parental cells with both CD44 and CD24 overexpression; CSC: TW01 cancer stem cells isolated by behavior selection. (*: *p*<0.05; **: *p*<0.01).

To further investigate whether overexpression of CD44 and CD24 plays roles in reprogramming differentiated cancer cells to CSC-like phenotype, we ectopically expressed CD44 and CD24 in NPC parental cells. We found that co-expression of CD44 and CD24 in TW01 NPC cell lines resulted in the upregulation of several stemness genes including *Oct4*, *Nanog, Klf4* and *c-Myc*, as well as the EMT-related genes *Twist* and *Snail* (Figure 6A). Overexpression of CD44 and/or CD24 significantly elevated the capacities of spherogenesis and soft agar formation (Figure 6B and 6C). Additionally, therapeutic resistance was significantly augmented after co-expression of CD44 and CD24 (Figure 6D and 6E). Through the cell migration and invasion assay, NPC parental cells with co-overexpression of CD44 and CD24 exhibited significant migration and invasion capacity compared with parental cells and either CD44 or CD24 overexpressed cells (Figures 6F and 6G and Supplementary Figure S5A). Because CD44 expression is already high in parental NPC cells, we overexpressed CD44 or CD24 in the parental cells with CD44/CD24-deficient background to unequivocally validate the gene regulation with just CD44 or CD24 overexpression (Supplementary Figure S5B). Stemness genes were more strikingly upregulated by CD24 whereas the expressions of EMT regulators were relatively enhanced by CD44 (Supplementary Figure S5B). These results validated the functionality of CD44 and CD24 in conferring CSC properties in differentiated NPC cells to give rise to CSCs.

### Inhibition of STAT3 phosphorylation impeded CSC properties conferred by CD44 and CD24 overexpression

**Figure 7:**
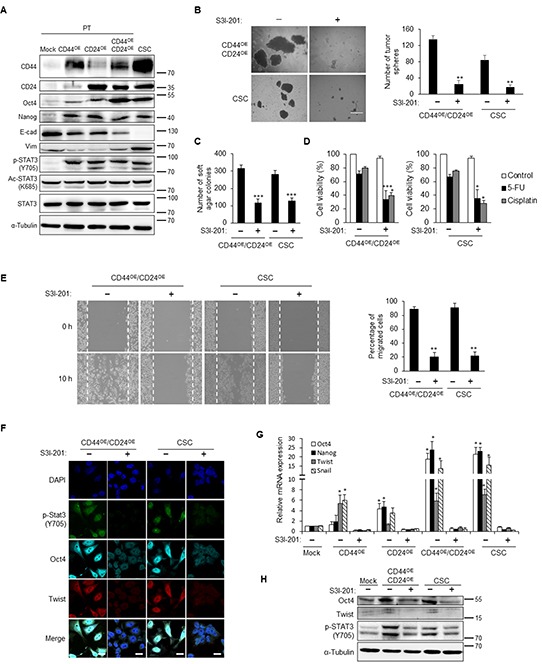
STAT3 inactivation blunts the CD44/CD24-induced CSC properties **A.** Stemness (Oct4 and Nanog) and mesenchyme (vimentin) markers were up-regulated in either CD44 or CD24 or co-overexpressed TW01 parental cells and CSCs. Phosphorylated STAT3 (p-STAT3) was concomitantly up-regulated, while acetylated STAT3 (Ac-STAT3) or STAT3 did not show significant change in these cells. α-tubulin served as internal control. **B.** Left images indicated the sphere morphology of TW01 CD44/CD24 co-overexpressed cells (CD44^OE^/CD24^OE^) and CSCs with or without S3I-201 treatment. Right plot shows that the numbers of tumor spheres of these two cells were significantly decreased upon S3I-201 treatment. **C.** The numbers of soft agar colonies of TW01 CD44/CD24 co-overexpressed cells and CSCs were significantly decreased under administration of S3I-201. **D.** Treatment with S3I-201 enhanced the sensitivity of TW01 CD44/CD24 co-overexpressed cells and CSCs to cisplatin or 5-FU. S3I-201 only did not cause significant cytotoxic effect. Relative percentages of viable cells of indicated cells were compared to the basal levels of CD44^OE^/CD24^OE^ or CSC cells without any treatments. **E.** S3I-201 reduced the migration capacity of TW01 CD44/CD24 co-overexpressed cells and CSCs. Right panel showed the quantified percentage of migrated cells at 10 hours post-wounding. **F.** Suppression of Oct4 and Twist expression was shown in TW01 CD44/CD24 co-overexpressed cells and CSCs after S3I-201 treatment. Scale bars indicate 20 μm. **G.** Stemness (Oct4 and Nanog) and EMT-associated (Twist and Snail) genes were reduced when treated with S3I-201 in either CD44 or CD24 or co-overexpressed cells and CSCs as measured by qPCR analysis. Results were normalized with the mRNA expression level of *18S rRNA* and compared with parental NPC cells. **H.** Western blot indicates the downregulation of Oct4 in TW01 CD44/CD24 co-overexpressed cells and CSCs. α-tubulin served as internal control. These results are representative of 3 independent experiments. Mock: TW01 parental cells with mock-infected; CD44^OE^: TW01 parental cells with CD44 overexpression; CD24^OE^: TW01 parental cells with CD24 overexpression; CD44^OE^/CD24^OE^: TW01 parental cells with both CD44 and CD24 overexpression; CSC: TW01 cancer stem cells isolated by behavior selection. (*, *p*<0.05; **, *p*<0.01; ***, *p*<0.001).

CD44 and CD24 were unveiled to regulate STAT3 activity by phosphorylation or acetylation of STAT3 to promote downstream signaling cascade. To explore the major downstream mediator of CD44 and CD24, we evaluated the phosphorylation or acetylation pattern of STAT3 in CD44 and CD24 co-expressed NPC cells. As concomitant upregulation of stemness and EMT factors, phosphorylated STAT3 was increased in cells when either CD44 or CD24 were ectopically expressed but not in its parental cells (Figure 7A and Supplementary Figure S5C). However, CD44 or CD24 overexpression did not affect the total form of STAT3 or acetylation form of STAT3 (Figure 7A and Supplementary Figure S5C). Translocation of phosphorylated STAT3 into the nucleus was also facilitated in either CD44 or CD24 or both overexpressed cells (Supplementary Figure S5D). These results demonstrated the positive correlation of phosphorylated STAT3 in CD44 and CD24 mediated reprogramming of NPC.

Accordingly, we hypothesized that CD44 and CD24 transcriptionally regulated CSC properties through STAT3 phosphorylation. To examine whether reprogramming of parental cells by CD44 and CD24 is STAT3 dependent, we attenuated the STAT3 activation of CSCs by a STAT3 inhibitor (S3I-201). After treatment with S3I-201, phosphorylated STAT3 was decreased in NPC CSC in a dose dependent manner, whereas total form of STAT3 expression was not affected (Supplementary Figure S6A). S3I-201 was also verified to inhibit the phosphorylation status of STAT3 under interleukin-6 (IL-6) stimulation (Supplementary Figure S6B). We found that inhibition of STAT3 phosphorylation significantly reduced the spherogenesis and soft agar formation in CD44 and CD24 co-overexpressed cells and CSCs (Figure 7B and 7C). Likewise, inhibition of STAT3 phosphorylation reduced the chemoresistance capacity in CD44 and CD24 co-overexpressed cells as well as NPC CSCs (Figure 7D and Supplementary Figure S6C). Moreover, treatment of S3I-201 significantly decreased the migration capacity in NPC cells with CD44 and CD24 overexpression and NPC CSCs (Figure 7E and Supplementary Figure S6D). Overexpression of CD44 and CD24 led to extensively upregulation of stemness and EMT related genes, whereas treatment with S3I-201 resulted in abolishment of these effects (Figures 7F–7H). These results substantiated that STAT3 activation is required in CD44 and CD24 mediated induction or maintenance of stemness and EMT in NPC CSCs.

## DISCUSSION

CSCs are considered as the main impediment in current cancer therapy. They can harbor many malignant traits, including resistance to therapy, recurrence, and metastasis. Unfortunately, it is difficult to isolate and enrich this rare sub-population of cancer cells from heterogeneous tumor masses without knowing their specific biomarkers. Therefore, we aimed to obtain the NPC CSCs with potent CSC properties first and then unveil which markers display on these CSCs. We designed the current isolation strategy using three distinctive behaviors of CSCs and this behavior selection process can serve as a platform to locate CSCs with unknown surface markers [30]. Accordingly, we found that these NPC CSCs possessed high levels of CD44 and CD24 surface proteins (Figure 1). The combined biomarkers CD44 and CD24 have been identified as CSC surface markers in breast, prostate, pancreatic, and colorectal cancers [31–33]. CD44^+^/CD24^−^ cells show CSC characteristics in breast and prostate cancers [31, 33]. However, CD44^+^/CD24^+^ cells, not CD44^+^/CD24^−^ cells, harbor CSC capacity in pancreatic and colorectal cancers [32, 33]. In our studies, the results revealed that CD44^high^/CD24^high^ sufficiently represents CSC surface markers in NPC. Pathological findings on paraffin-embedded primary NPC tumors revealed that CD44 is expressed even in early tumors, and then gradually increases in advanced tumors (Figure 2A). Different from CD44, CD24 was predominately expressed in advanced NPC tumors (Figure 2A), which implies that CD24 may be involved in advanced tumor progression.

Thus far, the mechanistic link between stemness and EMT has remained elusive. In this study, we found that ectopically expressed EMT regulator Twist in NPC led to the upregulation of the four stemness factors, and vice versa (Figures 3A–3C). This indicates a reciprocal regulation of stemness and EMT. Intriguingly, we found that CD44 and CD24 are essential in this reciprocal regulation in NPC CSCs. Down-regulation of CD44 and CD24 made NPC CSCs loss the expression of self-renewal markers (Figure 4A, Supplementary Figure S3A) and exhibit altered expression of many stemness and EMT markers (Figures 4C and 4D, Supplementary Figure S3B). The up-regulation of stemness genes was also blunted in Twist-iCSCs (Supplementary Figure S3B). In addition, depletion of CD44 and CD24 also reversed the EMT in 4F-iCSCs (Supplementary Figure S3B). These results have substantiated that CD44 and CD24 can serve as prerequisite bridges between stemness and EMT. Behavioral observation further depicted the critical roles of CD44 and CD24 in maintaining the CSC characteristics such as resistance to therapy, self-renewal capacity, tumor initiation capacity, and metastatic potential in NPC CSCs (Figures 5 and 6, and Supplementary Figures S4 and S5A).

CD44 and CD24 appear to be of vital importance in maintaining stemness and EMT as uncovered by knockdown of CD44 and CD24. However, it is particularly worth mentioning that CD44 and CD24 seem to modulate stemness and EMT differentially. Knockdown of both CD44 and CD24 could suppress the EMT process and stemness of CSCs. However, CD44 knockdown manifested higher efficacy to reverse the EMT process, while CD24 knockdown significantly suppressed the expression of stemness genes (Figures 4C–4E, Supplementary Figure S3B). Consistently, overexpression of CD44 specially stimulated the expression of EMT-related genes, while CD24 overexpression particularly upregulated the expression of stemness-related genes (Figure 6A and Supplementary Figure S5B). Behavioral observation also depicted the different roles of CD44 and CD24 in acquisition of the malignant traits of CSCs. We found that CD44 knockdown reduced the invasion capacity to a greater extent in CSCs compared with CD24 knockdown (Figure 5F, Supplementary Figure S4E). Compared with CD44 knockdown, depletion of CD24 notably inhibited more colony formation and spherogenesis capacity (Figures 5A and 5B, Supplementary Figures S4A and S4B). Loss of CD24 expression significantly abolished the resistance to chemotherapeutic agents and irradiation (Figures 5C and 5D, Supplementary Figures S4C and S4D). In line with loss-of-function assay, gain of CD24 function imparted more colony formation capacity in tumor sphere, soft agar assay, and therapeutic resistance in comparison with CD44 overexpression (Figures 6B–6E). In addition, these effects of knockdown of CD24 in CD44^high^/CD24^high^ NPC CSCs were similar with LMP1-induced CD44^high^/CD24^low^ NPC cells [19]. LMP1-induced CD44^high^ NPCs with low expression of CD24 manifested reduced expression of some stemness genes and low percentage of side population cells [19]. These results may substantiate the indispensable role of CD24 in maintaining CSC properties in NPC. Through manipulation of CD44 and CD24, we found that CD44 and CD24 may have different contribution in regulating stemness and EMT. Nevertheless, co-expression of CD44 and CD24 in NPC cells shared the most similarity with CSCs in gene expressions and behaviors (Figure 6), implying that CD44 and CD24 must pull together to boost the expression of stemness and EMT to achieve CSC properties.

CD44 and CD24 are disclosed to have common connections with STAT3 activity [18, 34]. STAT3 can be activated through acetylation or phosphorylation [35–37]. We found that phosphorylated STAT3 (Y705), not acetylated STAT3, was upregulated in CSCs and parental cells transduced with CD44 or CD24 (Figure 7A and Supplementary Figure S5C). STAT3 has a tyrosine phosphorylation site at position 705 (Y705) and a serine phosphorylation site at position 727 (S727) [35]. Y705 of STAT3 can be phosphorylated by various tyrosine kinases, such as Janus kinase (JAK), epidermal growth factor (EGFR), vascular endothelial growth factor receptor (VEGFR), and platelet derived growth factor receptor (PDGFR) [38]. STAT3 can also be phosphorylated at S727 by mitogen-activated protein kinases (MAPK), protein kinase Cδ (PKCδ), mTOR, and NLK, but the roles of phosphorylated S727 remains controversial.

STAT3 protein participates in diverse biological functions in normal and tumor cells [39–44]. Furthermore, the JAK/STAT3 signaling pathway correlates closely with prostate CSCs and required for tumor initiation [45]. In ovarian cancer cells, blockage of JAK/STAT3 signaling pathway hammers out the CSC traits [46]. Recent studies also demonstrated that STAT3 was a key regulator that maintains breast CSC subpopulation [47]. To unveil whether the regulation of CSC properties in CD44 and CD24 overexpressed NPC cells is STAT3 dependent, we utilized a STAT3 inhibitor (S3I-201) to examine the correlation of phosphorylated STAT3 and CSC characteristics. CSC behavior assays demonstrated that S3I-201 counteracted the effects of CD44 and CD24 (Figures 7B–7E and Supplementary Figures S6C and S6D). These results substantiated that CD44 and CD24 transcriptionally regulated CSC properties in a STAT3-dependent manner. STAT3 constitutive activation is found in various human cancers and several STAT3 inhibitors have been shown to act as effective cancer cures in preclinical studies. Consequently, STAT3 inhibition may serve as a novel approach to enhancing CSC targeted therapy. Besides, since CD44 and CD24 do not have kinase activity, they must require other kinase to activate STAT3. Previous studies uncovered that CD24 is able to phosphorylate STAT3 at Y705 through Src [18]. CD44 also can interact with JAK2 and STAT3 to activate STAT3 in breast cancer [34]. Hence, further studies are required to investigate which kinase is required for CD44 and CD24 to activate STAT3 in NPC CSCs.

Taken together, we identified that CD44 and CD24 are not merely physical markers but are functionally capable of driving stemness and EMT in CSCs. Our findings have revealed that CSCs utilize the CD44 and CD24 surface markers to orchestrate the CSC characteristics in a delicate manner. CD44/CD24/STAT3 axis also offers an imperative mechanistic link between stemness and EMT in the CSCs. The underlying mechanism of the functions of these CSC surface markers warrants further investigation. The information thus obtained may lead to a wealth of novel therapeutic targets, which may be useful in the development of anti-CSC targeted therapy for more effective treatments of cancer patients.

## MATERIALS AND METHODS

### Cell culture

Three human NPC cell lines, TW01, TW06, and HONE-1 were generously provided by Dr. Chin-Tarng Lin (National Taiwan University, Taipei, Taiwan) and maintained according to published protocols described previously [48, 49]. All primary NPC samples or tissue sections were obtained from the Department of Pathology or Department of Otorhinolaryngology, Taipei Veterans General Hospital. Written informed consent approved by the Institutional Review Board of Taipei Veterans General Hospital (VGHIRB No.:98-02-36A) was obtained from each of the patients. All NPC cell lines were maintained in complete Dulbecco's Modified Eagle Medium (DMEM), supplemented with 1% sodium pyruvate, 1% non-essential amino acids, 1% penicillin-streptomycin and 10% fetal bovine serum (Thermo Fisher Scientific Inc., Waltham, MA) at 37°C with humidified 5% CO_2_. For inactivation of STAT3, cells were treated overnight with S3I-201 (150 μM, Sigma-Aldrich Chemical Co., St. Louis, MO). Viable cells were used for further evaluation. To validate the effect of S3I-201, cells were also treated overnight with 50 ng/ml human interleukin-6 (Thermo Fisher Scientific Inc.).

### Derivation of CSCs by behavior selection

To obtain a high ratio of CSCs from NPC parental cells, we first developed a behavior selection method as described previously [30]. Briefly, the parental NPC cells were isolated through irradiation selection, spheroid formation, and side population selection. Firstly, radioresistant clones were picked following four rounds of 11 Gy irradiation at 37.9 mGy/s by a Rad Source RS 2000 X-ray biological irradiator (Rad Source Technologies, Inc., Suwanee, GA), and the radioresistant cells were then subjected to tumor sphere selection. Cells were seeded into a petri-dish coated with 0.4% soft agar supplemented with serum-free DMEM. The soft surface prevented the cells from attaching to the dish and initiated spheroids after 10 days. Lastly, CSCs were isolated with side population assay by the cells from tumor spheres. The cell density was adjusted to 10^6^ cells/mL with DMEM containing 2% FBS and 5 μM Hoechst 33342 (Sigma-Aldrich) for 90 min at 37°C. After washing twice with PBS, propidium iodide (PI) (2 μg/mL, Sigma-Aldrich) staining was used to exclude dead cells. Cells were maintained at 4°C in the dark and then subjected to isolation by a BD FACSAria Flow Cytometer (BD Biosciences, San Jose, CA). The side population gating requires control experiments with the ATP-binding cassette (ABC) transporter inhibitor. As a control group, cells were incubated with fumitremorgin C (10 μM FTC, Sigma-Aldrich) for 30 min at 37°C prior to and throughout Hoechst dye staining. FTC would block with the ABC transporters from extruding Hoechst dye. The side population cells would display high fluorescence when adding FTC to block the efflux of Hoechst dye. We then compared the pattern of cells with or without the treatment of FTC to designate and isolate side population cells.

### Survival assay

Survival fractions were compared between the control and the test samples under 0, 2, 4, 6, 8, 10, and 12 Gy irradiation. The seeding number of cells depended on the dose of irradiation. These cells were cultured for 10 days and the numbers of surviving colonies were counted. After fixation with 3:1 ratio of methanol and glacial acetic acid, the colonies were stained with 2% crystal violet. Colonies consisting of 50 cells or more were counted under a phase-contrast microscope and the surviving fraction was determined. The surviving fraction was calculated using the number of colonies divided by the number of cells seeded, and corrected with the plating efficiency. Plating efficiency was calculated by dividing the number of colonies with the number of cells seeded.

### Flow cytometry for cell surface marker analysis or sorting

Cells were trypsinized and stained with anti-CD44-FITC (BD Bioscience, San Jose, CA) and anti-CD24-PE (BD Biosciences) antibodies. Rat anti-mouse immunoglobulin G (IgG)-FITC and rat anti-mouse IgG-PE (BD Biosciences) were used as isotype controls. The cells were incubated on ice for more than 30 min and washed twice before analysis by Cytomics FC500 Flow Cytometry (Beckman Coulter, Inc., Fullerton, CA) or sorting by a set of BD FACSAria Flow Cytometer (BD Biosciences). To perform dispersal and FACS sorting of *in vivo* tumors, tumor masses were processed within 1 hr after they had been surgically excised. To maximize the purity of tumor tissue, areas of fat, non-tumor, or necrotic tissue were discarded. We then incubated the tumors in DMEM containing 4 mg/ml type III collagenase (Worthington Biochemical Corp, Lakewood, NJ) for 1 hr at 37°C with 5% CO_2_. The trypan blue exclusion test is applied to determine the number of viable cells in the extracted cells. The percentage of viable cells after dissociation is around 85-95. Cells that had the highest expression of CD44 and CD24 (less than 5%) in the CD44^+^/CD24^+^ subpopulation were named CD44^high^/CD24^high^ cells while cells that had the lowest expression of CD44 and CD24 (less than 5%) in the CD44^−^/CD24^−^ subpopulation were named CD44^low^/CD24^low^ cells.

### Tumorigenicity assay

Tumorigenicity was evaluated after injecting cells (100, 500, 10^3^, and 10^4^) into the sub-renal capsule of 7-8 week-old NOD/SCID mice. The tumor incidence was monitored for 6 weeks, and the distribution of GFP positive tumors was observed by illuminating with Youlum Sky-blue II epifluorescent light (Youlum Inc., New Taipei City, Taiwan).

### Immunohistochemistry staining and evaluation

Paraffin sections of formalin-fixed slides were deparaffinized in xylene and rehydrated in ethanol. Antigen retrieval was performed by placing slides in citric acid (pH 6.0) at 120°C in a Retriever for 5 minutes. EnVision™ G2 Doublestain System, Rabbit/Mouse (DAB+/Permanent Red) (DAKO, Denmark) were applied to detect CD44 and CD24. Primary antibodies (Abcam, Cambridge, MA) for CD44 and CD24 were detected with diaminobenzidene (DAB) and Permanent Red, respectively. Hematoxylin was used for nuclear stain. The slides were then scanned by an Aperio Scanscope CS scanner (Aperio Techologies, Inc., Vista, CA). Immunoreactivity was semi-quantified by a method that determines the score based on combined intensity and the percentage of cells with positive stain [50].

### Statistical analysis

All data are presented as mean ± SD. Statistical analysis with Student's *t* test was performed by SPSS window version 20 software. A difference between groups with *p*<0.05 was considered significant.

The detailed technical information is available in Supporting Information.

## SUPPLEMENTARY MATERIALS AND METHODS, SUPPLEMENTARY FIGURES AND TABLES




