# Supplementary material for: CD44 and CD24 coordinate the reprogramming of nasopharyngeal carcinoma cells towards a cancer stem cell phenotype through STAT3 activation
Source: Oncotarget. 2016 Aug 8;7(36):58351–66. doi: 10.18632/oncotarget.11113 (PMC5295435; doi:10.18632/oncotarget.11113)
Supplement: Supplementary file 1 [file oncotarget-07-58351-s001.pdf]

CD44 and CD24 coordinate the reprogramming of nasopharyngeal carcinoma cells towards a cancer stem cell phenotype through STAT3 Activation

SUPPLEMENTARY MATERIALS AND METHODS

Irradiation selection

A total of 5×10^3 cells were seeded into 6-well cell culture plates in complete DMEM. After two hours of cell seeding, various doses from 0 to 12 Gy were given to determine the sublethal dose for each cell line. Irradiation was delivered at room temperature at 37.9 mGy/s using Rad Source RS 2000 X-ray biological irradiator (Rad Source Technologies, Inc., Suwanee, GA). The range of sublethal dose was 8-11 Gy in NPC cell lines. To establish radioresistant clones, three different irradiation doses were used to execute irradiation selection, including four rounds of 8 Gy, four rounds of 11Gy, and 8 Gy, 9 Gy, 10 Gy, 11 Gy, in each round of irradiation. After four rounds of irradiation selection, a single colony selection was performed to obtain homogeneous radioresistant clones. Combining the four rounds of irradiation and single colony selection, NPC radioresistant clones were obtained that had a radioresistant phenotype verified by survival fraction assay.

Tumor sphere formation assay

1×10^4 cells were trypsinized into individual cells and seeded into 1.2% soft agar coated petri-dishes with serum-free DMEM medium supplemented with 1% sodium pyruvate, 1% NEAA and 1% antibiotics-antimycotics solution. The soft surface rendered the cells unable to attach and they subsequently formed tumor spheres after a few days in suspension. The number of tumor spheres was counted after 10 days.

Side population selection

The cell concentration was adjusted to 10^6 cells/ml and incubated with 1 µl verapamil (50 µM, Sigma-Aldrich, St. Louis, MO) or fumitremorgin C (FTC, 10 µM, Sigma-Aldrich) to block the ABCG2 transporter. Hoechst 33342 (5 µM, Sigma-Aldrich), the DNA binding dye, was then added at a final concentration of 5 µg/mL and the cells were incubated in a 37°C incubator for 90 min. The tube was shaken every 15 min to avoid cell precipitation. After 90 min, the cells were washed twice with PBS, 2 µg/ml propidium iodide (PI) was then added, and the cells were kept at 4°C in the dark before sorting using a BD FACSAria Flow Cytometer (BD Biosciences). After UV488 laser excitation, major cell populations containing Hoechst 33342 showed blue and red fluorescence light. A subset of side population cells pumped out the Hoechst dye *via* ABC-transporters and demonstrated low fluorescence expression. These side population cells exhibited high fluorescence when treated with verapamil or FTC to block the efflux of fluorescence. The patterns were compared with or without treatment with verapamil or FTC to isolate side population cells.

Plasmids, virus production and infection

Human Twist cDNA was subcloned into pLV-EF1a-IRES-Hyg lentiviral vector (BioSettia, San Diego, CA). The development of pSin-EF2-Oct4-Pur, pSin-EF2-Sox2-puro, pSin-EF2-Klf4-puro, pMXs-hc-Myc have been reported earlier [1–3]. CD44 and CD24 shRNA were cloned in pLKO.1-based lentiviral vector provided by the National RNAi Core Facility, Academia Sinica, Taiwan. We chose the CD44 and CD24 shRNA with the best knockdown efficiency to do further experiment; these were picked from three CD44 shRNAs and three CD24 shRNAs targeted against different regions of CD44 and CD24. Additionally, human CD44 cDNA was subcloned into pBABE-puro vector (Addgene, Cambridge, MA) and CD24 cDNA was purchased from GeneCopoeia (Germantown, MD).

A total of 10^6 293T cells were seeded on 100 mm dishes and cultured overnight. Utilizing PolyJet™ DNA *In Vitro* Transfection Reagent (SignaGen Laboratories, Rockville, MD), cells were transfected with the viral vectors. The medium was replaced with complete DMEM 24 hours post-transfection. The virus-containing supernatants of the transfectants were collected and filtered through a 0.45 µm pore size filter 48 hours post-transfection. Viral infection was achieved by adding virus-containing supernatant supplemented by 8 ng/ml polybrene to target cells. Antibiotic selection 24 hours post infection was subsequently employed.

Real-time RT-PCR analysis

Total RNA was extracted with TRIsure™ reagent (Bioline Reagents Ltd, London, UK). The concentration and purity of total RNA was determined using a NanoDrop ND-1000 Spectrophotometer (NanoDrop Technologies, Inc., Wilmington, DE). Real-time PCR was performed using the SensiFAST™ SYBR Hi-ROX Kit (Bioline) on the ABI StepOnePlus™ Real-Time PCR machine (Applied Biosystems, Foster City, CA). The PCR primer sequences are listed in Supplementary Table S1.

Western blot

Total cellular protein was extracted in complete RIPA complete RIPA containing protease inhibitor, and phenylmethylsulfonyl fluoride (PMSF) (all from Sigma-Aldrich). The protein assay kit (Bio-Rad, Hercules, CA) was used to determine protein concentrations using a UV-visible spectrophotometer U-3300 (Hitachi, Tokyo, Japan). Aliquots containing 50 μg of total protein from each sample were separated by electrophoresis on 8%, 10%, or 12% SDS-polyacrylamide gels and electro-blotted to BioTrace PVDF membranes (Pall Corporation, Port Washington, NY). After blocking with 4% non-fat dried milk, primary antibodies such as anti-α-tubulin (Abcam, Cambridge, MA), anti-Oct3/4 (Bioworld Technology, Suffolk, UK), anti-Sox2 (Cell Signaling, Boston, MA), anti-Klf4 (Abcam), anti-c-Myc (Cell Signaling), anti-Nanog (Sigma-Aldrich), anti-Twist (GeneTex, Irvine, CA), anti-Claudin-3 (Bioworld Technology), anti-Occludin (Invitrogen), anti-E-cadherin (BD **Biosciences**), anti-N-cadherin (BD **Biosciences**), anti-Vimentin (Sigma-Aldrich), anti-STAT3 (Cell Signaling Technology Danvers, MA), anti-p-STAT3 (Y705) (Cell Signaling), or anti-Ac-STAT3 (K685) (Cell Signaling), were used for hybridization in 4% non-fat dried milk; this was followed by incubation with appropriate secondary antibodies (Sigma-Aldrich). The protein bands were captured using a Luminescence Imaging System (Fujifilm, Tokyo, Japan).

Soft agar assay

The plates were first de-coated with 1.2% soft agar as a base. After the agar solidified, 1×10^4 cells were mixed with 0.4% soft agar and seeded on the base. The colonies grown in soft agar were counted after two weeks.

Wound healing migration assay

We utilized ibidi culture inserts (ibidi GmbH, Munich, Germany) for wound healing migration assay and performed according to the manufacture's protocol. The insert contains two reservoirs separated by a 500 μm thick wall. We placed the culture insert into a 24-well culture plate and added 1×10^4 cells into each reservoir for overnight culture. A 500 μm gap was created after removing the culture insert. The images of cell migration were captured by an EVOS FL Cell Imaging System (Thermo Fisher Scientific Inc.).

Transwell invasion assay

For invasion assay, we placed Millicell invasion chamber (8 μM pore size, Millipore, Darmstadt, German) with Matrigel (BD Biosciences) into a 24-well plate. In the upper compartment of the invasion chamber, 1×10^4 cells were seeded and filled with 200 μL serum-free DMEM. In the lower compartment of the invasion chamber, 600 μL complete DMEM with 10% FBS. After 24 hours incubation, the invasive cells located on the underside of the filter were fixed with 3:1 ratio of methanol and glacial acetic acid, stained with 2% crystal violet, and counted under a phase-contrast microscope.

Immunofluorescence staining

Cells were seeded on the cover slides for 24 hours and then fixed in 4% paraformaldehyde and permeabilized in 0.3% Triton X-100 in PBS. The fixed cells were then washed three times with PBS and blocked in FBS. The cells were incubated with primary antibodies such as anti-Oct3/4 (Cell Signalling), anti-Nanog (Cell Signaling), anti-Twist (GeneTex, Irvine, CA), anti-Occludin (Invitrogen), anti-E-cadherin (BD Biosciences), anti-N-cadherin (BD Biosciences), anti-Vimentin (Cell Signalling), anti-CD44 (Cell Signalling), anti-CD24 (Abcam), or anti-p-STAT3 (Y705) (Cell Signaling) at 4°C overnight, washed three times with PBS plus 0.1% Tween-20, and incubated with appropriate secondary antibodies (HiLyte Fluor™, AnaSpec, Inc. Fremont, CA) for 2 hours. The slides were mounted with Dapi-Fluoromount-G™ (SouthernBiotech, Birmingham, AL) after brief PBS washing. The control and test samples were photographed with the same exposure time using an Olympus FV-10i (Olympus Optical, Tokyo, Japan).

Immunoreactivity evaluation for immunohistochemistry staining

Immunoreactivity was semi-quantified by a method that determines the score based on combined intensity and the percentage of cells with positive stain according to a previous report [4]. Briefly, compared with the positive control, the same degree of staining intensity was graded as 2. When the intensity was weaker than the control, the cells were graded as 1, and when stronger than the control, they were graded as 3. Negative staining was 0. We then counted about 1000 NPC cells and the percentage of cells with each intensity grade was estimated. The staining score for CD44 and CD24 antibodies (Abcam) was defined as the sum of the percentage of positive cells with each intensity level multiplied by the intensity grade (e.g., a case with 40% grade 3, 40% scored 2, and 20% scored 1 staining would be scored as $40\times3+40\times2+20\times1=220$).

Cell viability assay

Cells were exposed to cisplatin (8 μM), 5-fluorouracil (5-FU; 10 μM), or irradiation (5 Gy and 10 Gy). After 2 days of culture, cells were washed with PBS and replenished with fresh medium containing 1×alamarBlue® cell viability assay reagent (AbD Serotec, Oxford, UK) in 37°C incubator for 2 hours. Fluorescence intensity, with the excitation wavelength at 538 nm and the emission wavelength at 590 nm, was measured using an Infinite® 200 multimode microplate reader (Tecan Group Ltd).

REFERENCES

1. Yu J, Vodyanik MA, Smuga-Otto K, Antosiewicz-Bourget J, Frane JL, Tian S, Nie J, Jonsdottir GA, Ruotti V, Stewart R, Slukvin II, Thomson JA. Induced pluripotent stem cell lines derived from human somatic cells. Science. 2007; 318:1917–1920.
2. Kitamura T, Koshino Y, Shibata F, Oki T, Nakajima H, Nosaka T, Kumagai H. Retrovirus-mediated gene transfer and expression cloning: powerful tools in functional genomics. Exp Hematol. 2003; 31:1007–1014.
3. Takahashi K, Tanabe K, Ohnuki M, Narita M, Ichisaka T, Tomoda K, Yamanaka S. Induction of pluripotent stem cells from adult human fibroblasts by defined factors. Cell. 2007; 131:861–872.
4. Koga F, Kawakami S, Fujii Y, Saito K, Ohtsuka Y, Iwai A, Ando N, Takizawa T, Kageyama Y, Kihara K. Impaired p63 expression associates with poor prognosis and uroplakin III expression in invasive urothelial carcinoma of the bladder. Clin Cancer Res. 2003; 9:5501–5507.

SUPPLEMENTARY FIGURES AND TABLES

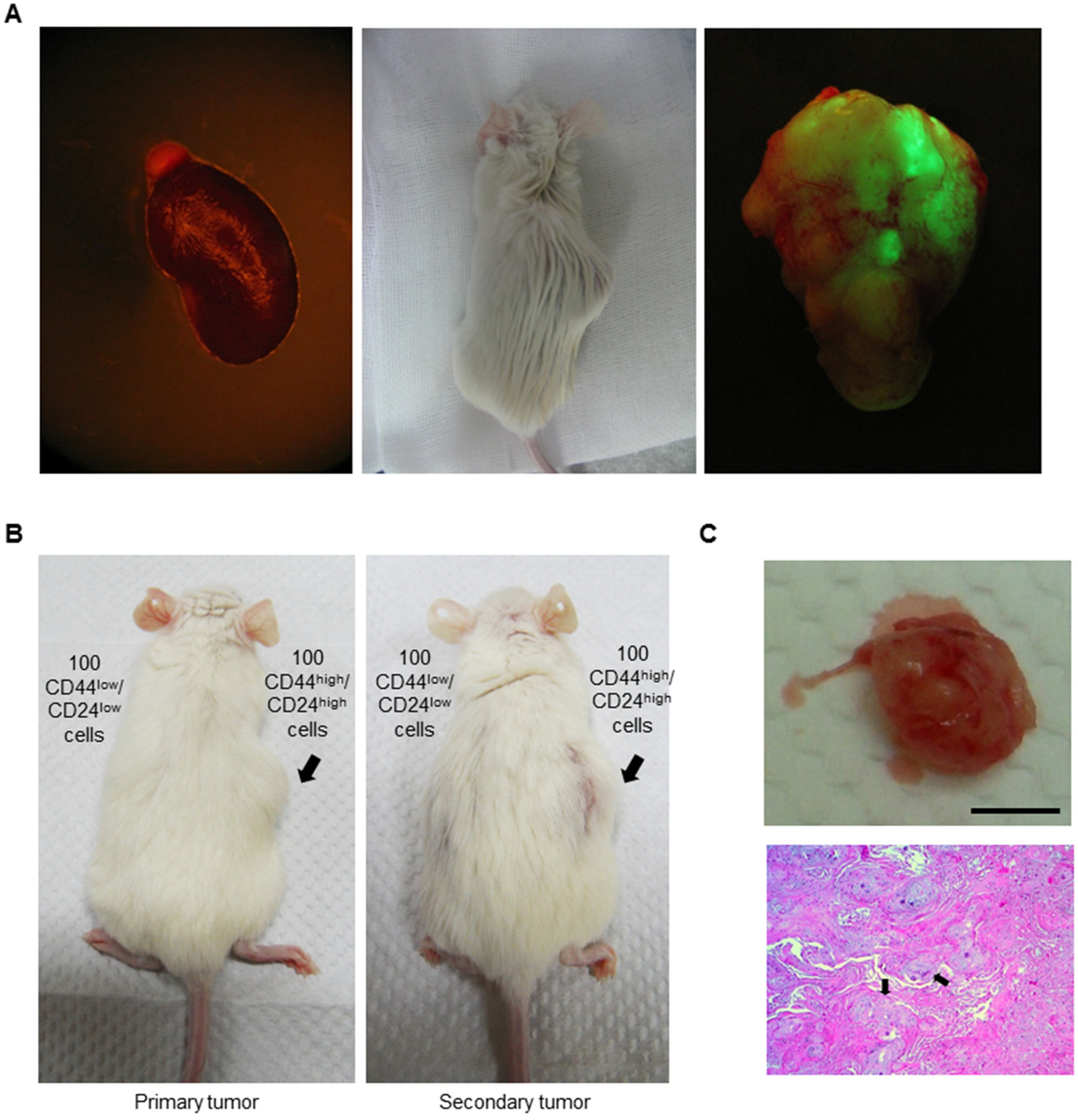

Supplementary Figure S1: CD44^{high}/CD24^{high} cells possess high tumorigenicity. A. A total of 500 GFP-lentivirus-infected TW01 CD44^{high}/CD24^{high} cells formed tumor in the sub-renal capsule (right side of mouse). However, 500 GFP-lentivirus-infected TW01 CD44^{low}/CD24^{low} cells did not form tumor (left side of the mouse). The fluorescent tumor cells were recognized by Sky-blue II epifluorescent light. **B.** A total of100 TW01 CD44^{high}/CD24^{high} cells initiated tumors in the primary and secondary NOD/SCID mice; whereas 100 TW01 CD44^{low}/CD24^{low} cells did not generate any tumor in the primary and secondary mice. Black arrows indicate the tumor masses. **C.** Upper image shows that CD44^{high}/CD24^{high} cells sorted from clinical NPC samples formed tumors in NOD/SCID mice. Scale bar indicates 5 mm. Lower image is the H&E stain of the tumor section. Tumor cells are clustered as indicated by the arrows.

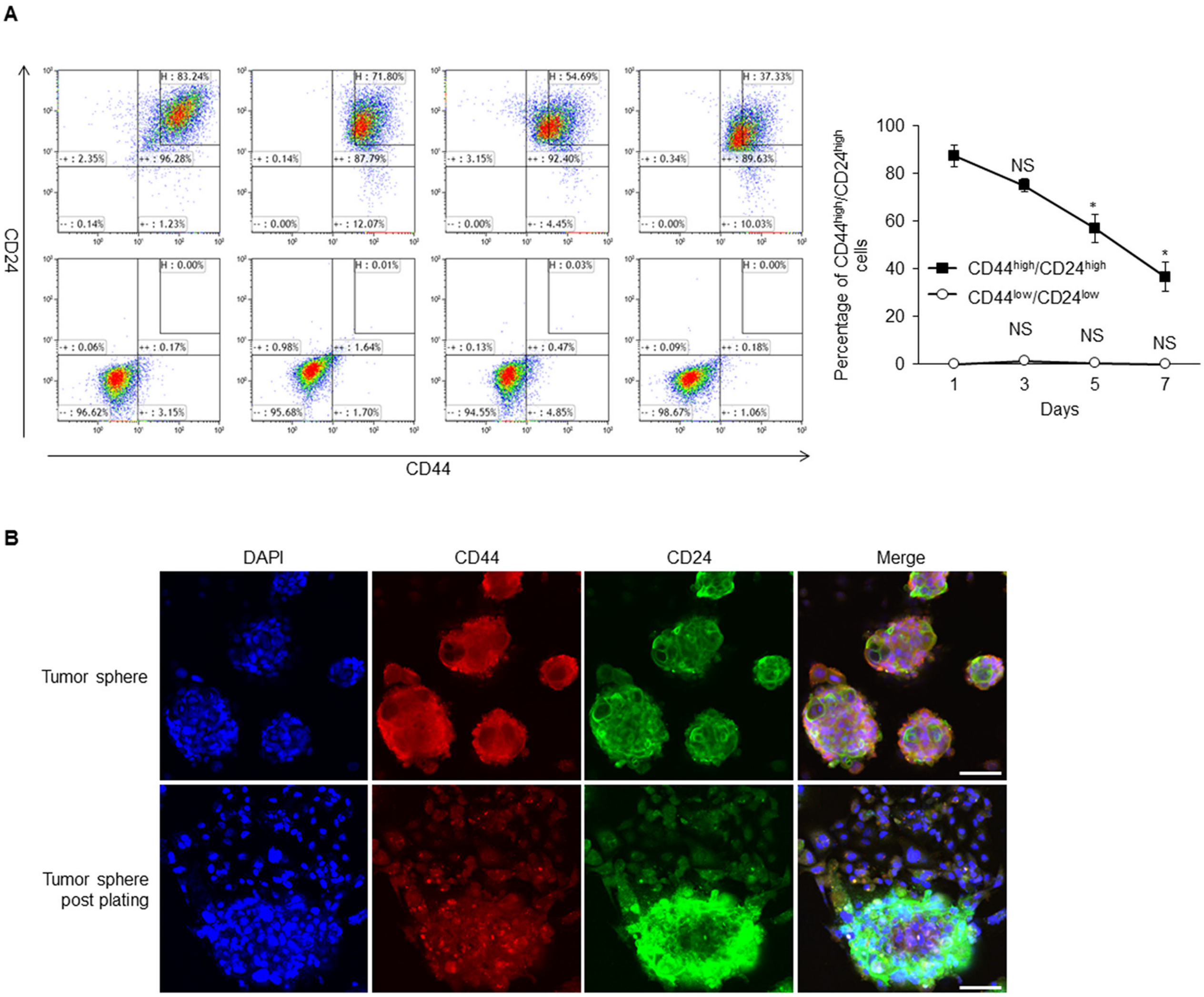

Supplementary Figure S2: Evaluation of the differentiation potential of CSCs. A. TW01 CD44^{high}/CD24^{high} cells underwent differentiation and resulted in decreasing number of CD44^{high}/CD24^{high} cells gradually as shown by dot plots (left) and quantitative data (right). The TW01 CD44^{low}/CD24^{low} cells merely divided into CD44^{low}/CD24^{low} cells that did not result in increasing CD44^{high}/CD24^{high} cells. The gating regions in left dot plots depicted the percentage of CD44^{high}/CD24^{high} cells. These results are representative of 3 independent experiments. (The percentages of CD44^{high}/CD24^{high} of indicated cells on day 3, 5, and 7 were compared with that of indicated cells on day 1; *, $p<0.05$; **, $p<0.01$; NS, not significant). **B.** Images of TW01 tumor spheres immunostained with antibodies against CD44 (red) and CD24 (green). The tumor spheres enriched CD44^{high}/CD24^{high} cells (top). After plating on the coated dish, cells within the tumor sphere began to spread out and reduce the expression levels of CD44 and CD24 (bottom). Scale bars indicate 100 μm.

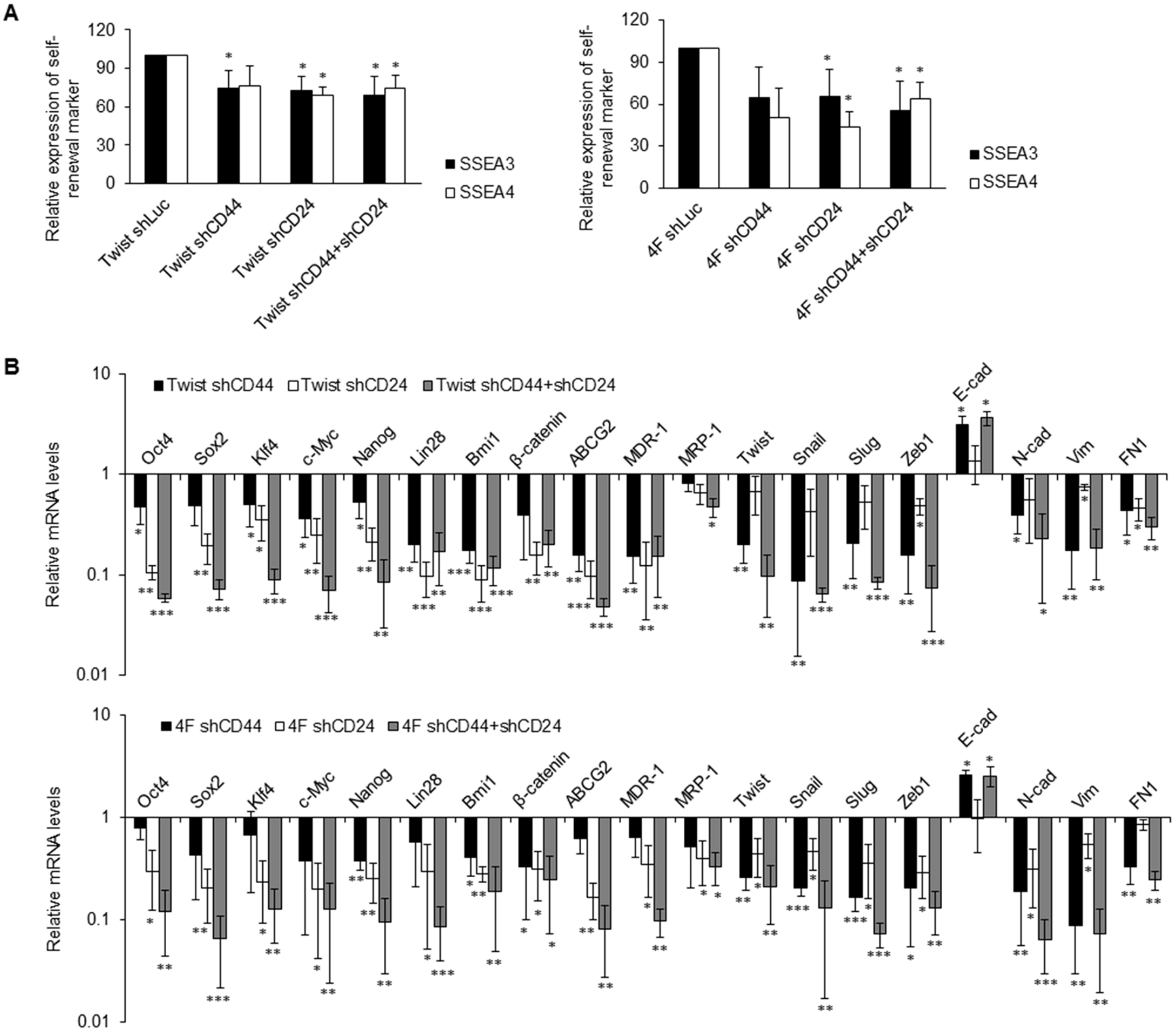

Supplementary Figure S3: Depletion of CD44 and CD24 suppresses stemness and EMT in CSCs. A. CD44 and CD24 knockdown resulted in the decrease of SSEA3 and SSEA4 expression in TW01 Twist-iCSCs and 4F-iCSCs as measured by flow cytometry. **B.** Knockdown of CD44 and CD24 altered the indicated genes in TW01 Twist-iCSCs and 4F-iCSCs as measured by qPCR. These results are representative of 3 independent experiments. (*, $p<0.05$; **, $p<0.01$; ***, $p<0.001$).

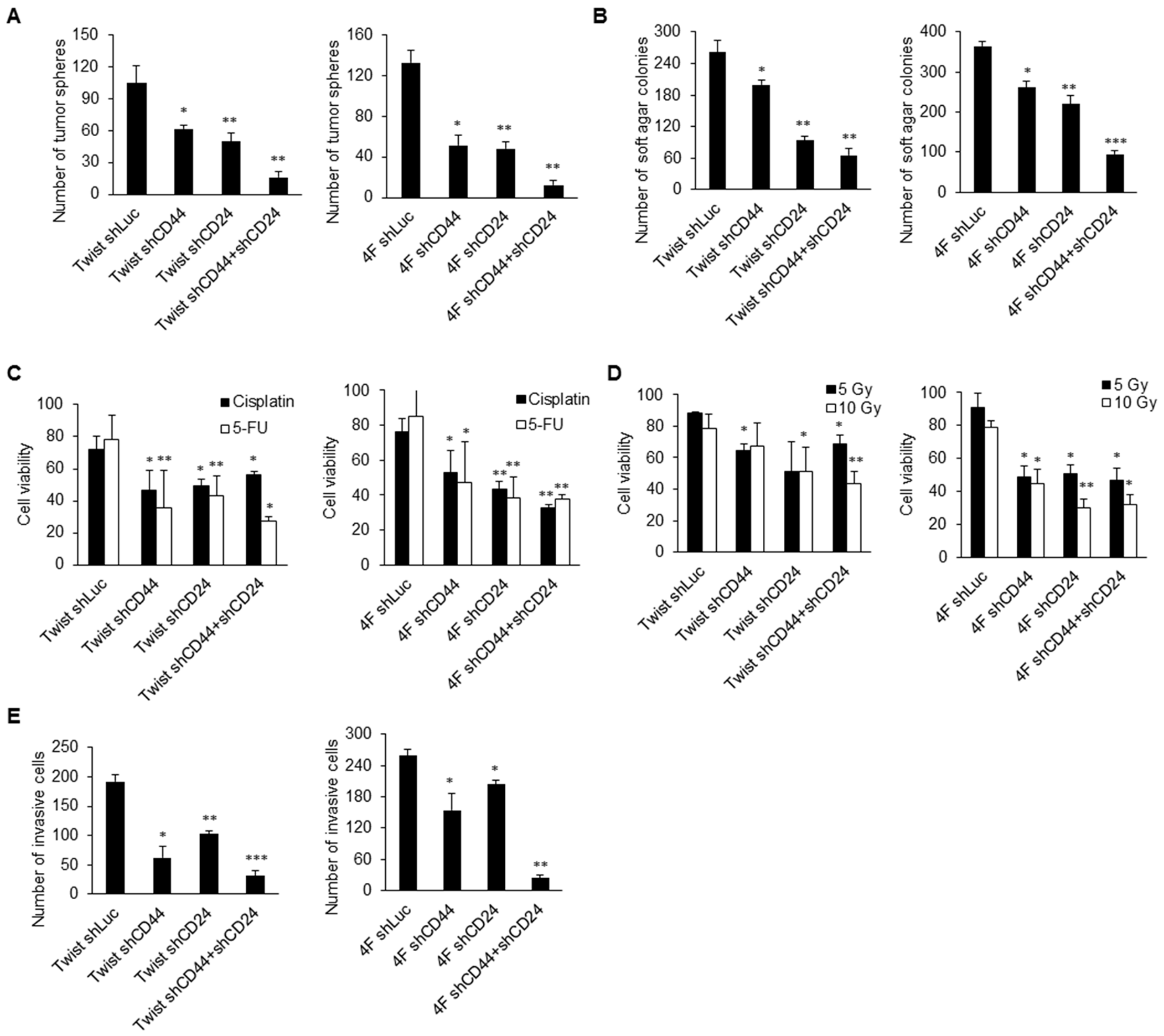

Supplementary Figure S4: Attenuation of aggressive phenotypes via knockdown of CD44 and CD24 in Twist-iCSCs and 4F-iCSCs. A. CD44 and CD24 knockdown TW01 iCSCs exhibited reduced colony formation in soft agar assay. **B.** Knockdown of CD44 and CD24 resulted in decrease of tumor sphere formation in TW01 iCSCs. **C.** Cell viability assay revealed that knockdown of CD44 and CD24 increased the sensitivity to cisplatin (8 μM) and 5-fluorouracil (5-FU; 10 μM) in TW01 iCSCs. **D.** The viability assay of TW01 iCSCs treated with 5 Gy and 10 Gy irradiation revealed that knockdown of CD44 and CD24 increased the radiosensitivity. **E.** Transwell invasion assay revealed the reduction of invasion capacity of TW01 iCSCs with CD44 and CD24 knockdown. These results are representative of 3 independent experiments. (*, p<0.05; **, p<0.01; ***, p<0.001).

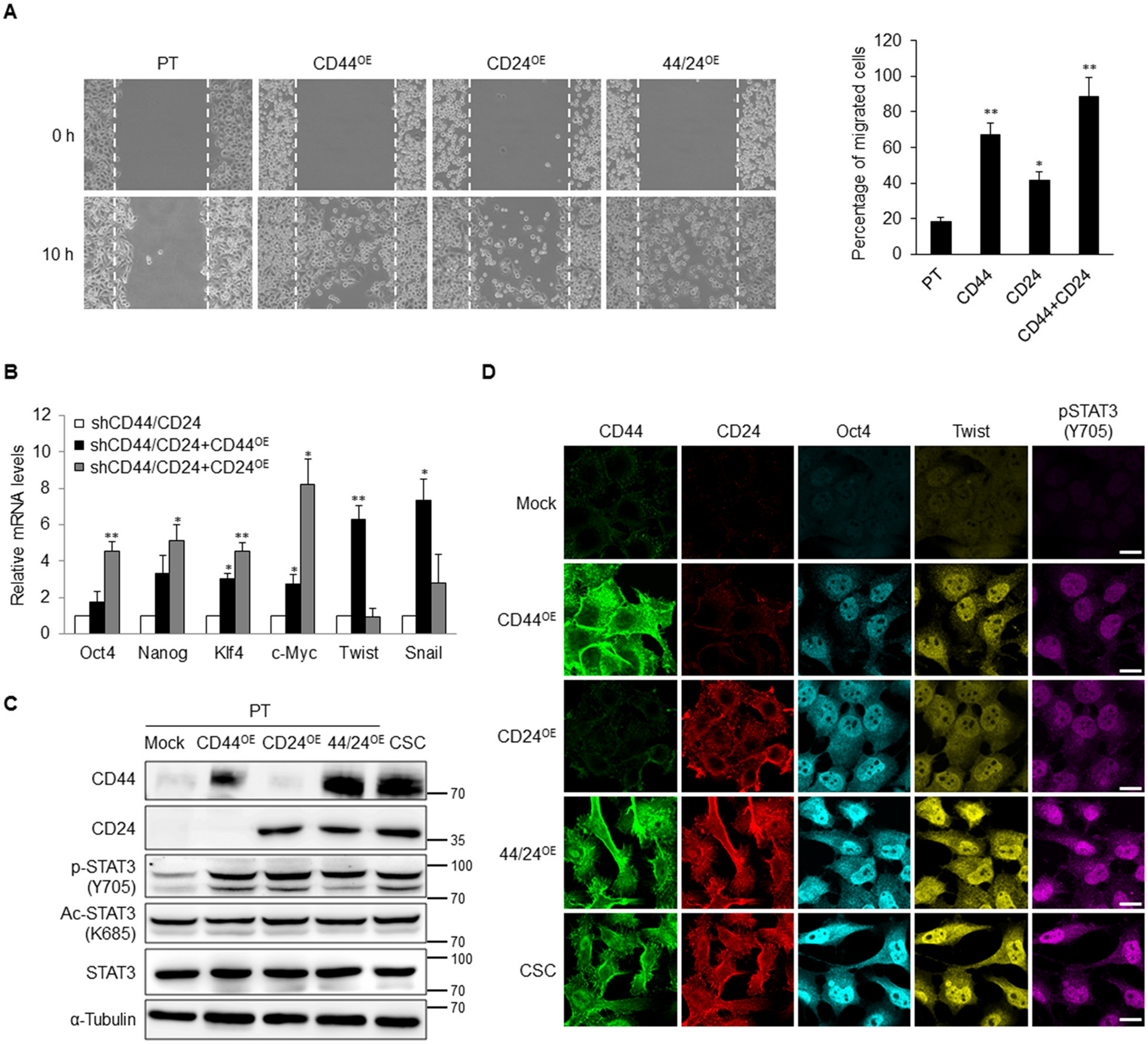

Supplementary Figure S5: Phosphorylation of STAT3 is positively correlated with overexpression of CD44 and CD24. **A.** HONE-1 CD44 and CD24 co-overexpressed cells possessed highest migration capacity compared with other groups. Right panel showed the quantified percentage of migrated cells at 10 hours post-wounding. **B.** Overexpression of CD44 or CD24 in TW01 parental cells with CD44 and CD24 knockdown altered the stemness and EMT expressions. Results were got through qPCR analysis, normalized with the mRNA expression level of *18S rRNA* and compared with parental NPC cells in a CD44/CD24-deficient background. **C.** P-STAT3 (Y705) were up-regulated, while Ac-STAT3 and STAT3 did not change in HONE-1 parental cells with CD44 or CD24 or both overexpressed cell. **D.** Overexpression of either CD44 or CD24 or both stimulate the expression of Oct4 (cyan fluorescence) and Twist (yellow fluorescence) as indicated by immunofluorescence staining in TW01 parental cells. p-STAT3 (purple fluorescence) can also be detected in nuclei. Scale bars indicate 20 μm. These results are representative of 3 independent experiments. (*, $p<0.05$; **, $p<0.01$).

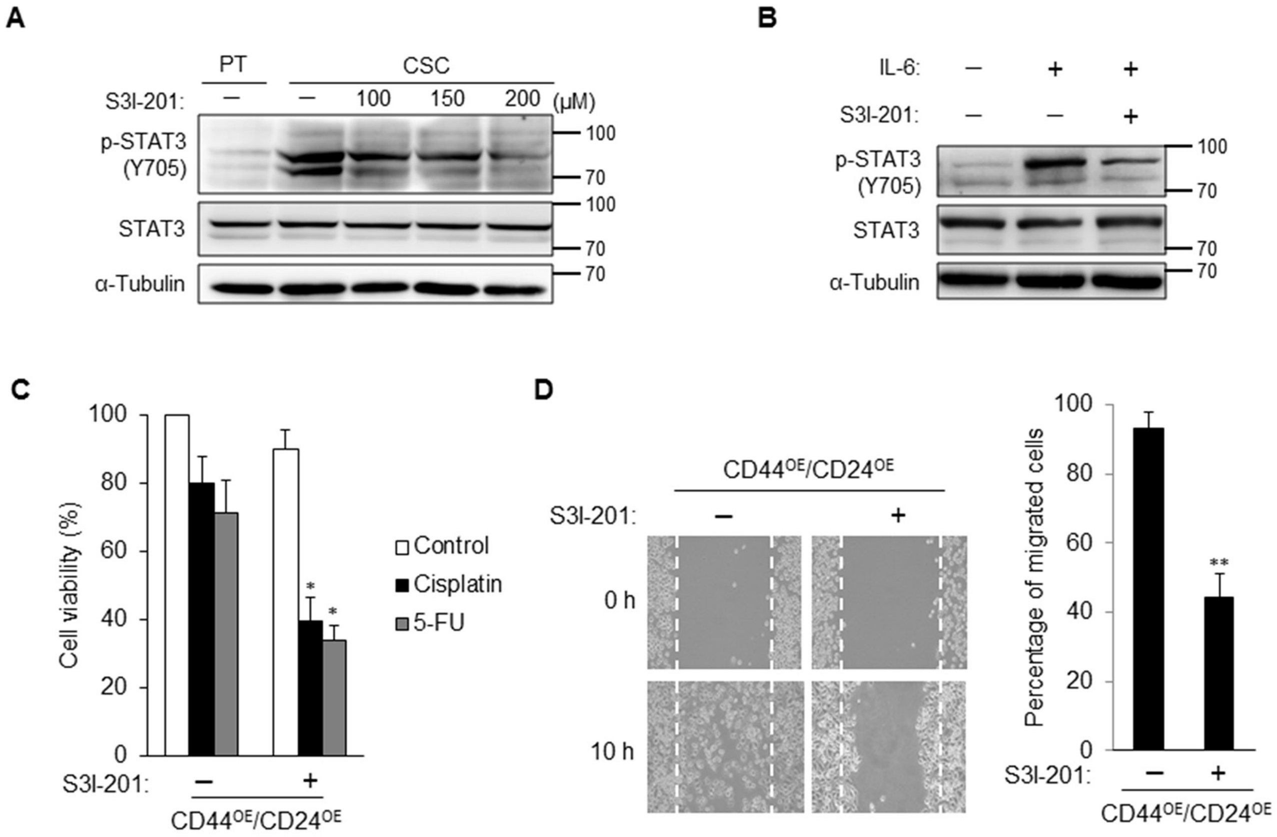

Supplementary Figure S6: CD44/CD24/STAT3 axis is essential in regulation of aggressive phenotypes in NPC cells. **A.** As detected by western blotting, level of phosphorylated STAT3 (p-STAT3) was increased in TW01 CSCs but this effect was abolished upon administration of S3I-201 in a dose-dependent manner. α-tubulin served as internal control. **B.** IL-6 inducing phosphorylation of STAT3 was aboligated by S3I-201. α-tubulin served as internal control. **C.** Treatment with S3I-201 enhanced the sensitivity of HONE-1 CD44 and CD24 co-overexpressed cells to cisplatin or 5-FU. S3I-201 only did not cause significant cytotoxic effect. Relative percentages of viable cells of indicated cells were compared to the basal levels of $CD44^{OE}/CD24^{OE}$ cells without any treatments. **D.** S3I-201 reduced the migration capacity of HONE-1 CD44 and CD24 co-overexpressed cells. Right panel showed the quantified percentage of migrated cells at 10 hours post-wounding. These results are representative of 3 independent experiments. (*,$p<0.05$; **, $p<0.01$).

Supplementary Table S1: Primer sequences.

See Supplementary File 1
