# Supplementary material for: CD44 and CD24 coordinate the reprogramming of nasopharyngeal carcinoma cells towards a cancer stem cell phenotype through STAT3 activation
Source: Oncotarget. 2016 Aug 8;7(36):58351–66. doi: 10.18632/oncotarget.11113 (PMC5295435; doi:10.18632/oncotarget.11113)
Supplement: Supplementary file 2 [file oncotarget-07-58351-s002.docx]

**Supplementary Table S1.** Primer sequences

| Primer | Sequence (5' to 3') |
| --- | --- |
| 18S rRNA-F | CTC AAC ACG GGA AAC CTC AC |
| 18S rRNA-R | CGC TCC ACC AAC TAA GAA CG |
| Oct4-F | GTG GAG AGC AAC TCC GAT G |
| Oct4-R | TGC TCC AGC TTC TCC TTC TC |
| Sox2-F | CGA GTG GAA ACT TTT GTC GGA |
| Sox2-R | TGT GCA GCG CTC GCA G |
| Klf4-F | CCG CTC CAT TAC CAA GAG CT |
| Klf4-R | ATC GTC TTC CCC TCT TTG GC |
| c-Myc-F | GGA ACG AGC TAA AAC GGA GCT |
| c-Myc-R | GGC CTT TTC ATT GTT TTC CAA CT |
| Nanog-F | ATT CAG GAC AGC CCT GAT TCT TC |
| Nanog-R | TTT TTG CGA CAC TCT TCT CTG C |
| Lin28-F | CCC CCC AGT GGA TGT CTT T |
| Lin28-R | CCC TCC TTC AAG CTC CGG |
| Bmi1-F | AAA TGC TGG AGA ACT GGA AAG |
| Bmi1-R | CTG TGG ATG AGG AGA CTG C |
| β-Catenin-F | CCA GCC GAC ACC AAG AAG |
| β-Catenin-R | CGA ATC AAT CCA ACA GTA GCC |
| ABCG2-F | CAT GTA CTG GCG AAG AAT ATT TGG T |
| ABCG2-R | CAC GTG ATT CTT CCA CAA GCC |
| MDR-1-F | TGG CAA AGA AAT AAA GCG ACT GA |
| MDR-1-R | CAG GAT GGG CTC CTG GG |
| MRP-1-F | GCT TCC TCT TGG TGA TAT TCG |
| MRP-1-R | GCA GTT CAA CGC ATA GTG G |
| Twist-F | GGA GTC CGC AGT CTT ACG AG |
| Twist-R | TCT GGA GGA CCT GGT AGA GG |
| Snail-F | CCT CCC TGT CAG ATG AGG AC |
| Snail-R | CCA GGC TGA GGT ATT CCT TG |
| Slug-F | GGG GAG AAG CCT TTT TCT TG |
| Slug-R | TCC TCA TGT TTG TGC AGG AG |
| Zeb1-F | ACT GCT GGG AGG ATG ACA GA |
| Zeb1-R | ATC CTG CTT CAT CTG CCT GA |
| E-cadherin-F | TGC CCA GAA AAT GAA AAA GG |
| E-cadherin-R | GTG TAT GTG GCA ATG CGT TC |
| N-cadherin-F | ACA GTG GCC ACC TAC AAA GG |
| N-cadherin-R | CCG AGA TGG GGT TGA TAA TG |
| Fibronectin-F | CAG TGG GAG ACC TCG AGA AG |
| Fibronectin-R | TCC CTC GGA ACA TCA GAA AC |
| Vimentin-F | GAG AAC TTT GCC GTT GAA GC |
| Vimentin-R | GCT TCC TGT AGG TGG CAA TC |
